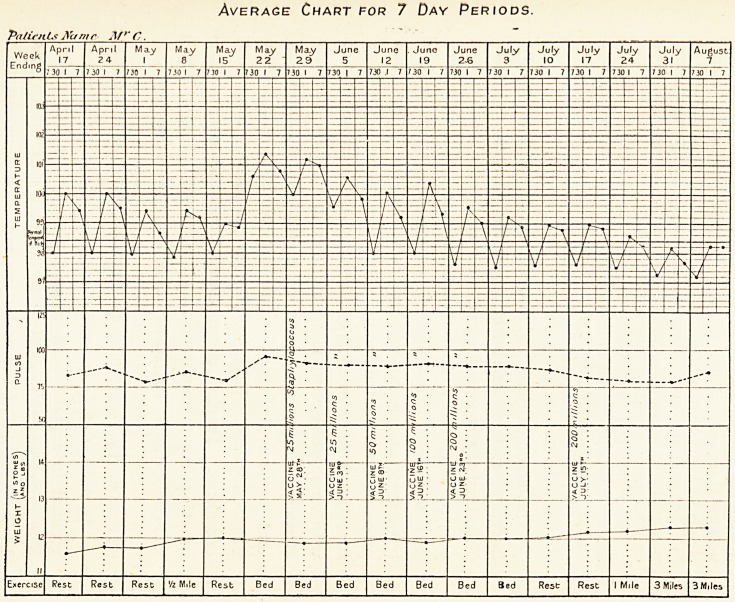# Mixed Infections in Pulmonary Tuberculosis, and Some General Observations on Treatment with Tuberculin
1A paper read at a discussion at a Meeting of the Bristol Medico-Chirurgical Society on May 13th, 1914.


**Published:** 1914-06

**Authors:** Noel Bardswell

**Affiliations:** Medical Superintendent, King Edward VII Sanatorium, Midhurst; Member of the Government Advisory Committee on Tuberculosis


### MIXED INFECTIONS IN PULMONARY TUBERCULOSIS, AND SOME GENERAL OBSERVATIONS ON TREATMENT WITH TUBERCULIN.[1]

BY

NOEL BARDSWELL, M.D. Edin., M.R.C.P. Lond. and Edin., F.R.S.E.,

*Medical Superintendent, King Edward VII Sanatorium, Midhurst; Member of the Government Advisory Committee on Tuberculosis.*

---

### ON MIXED INFECTION IN PULMONARY TUBERCULOSIS.

FOR some years Dr. Radcliffe, the resident Pathologist at King Edward VII Sanatorium, has been working on this subject, and the results of his observations are detailed in his Weber-Parkes Prize Essay for 1912.

He would distinguish between a true secondary infection and an accompanying or intercurrent infection. By the term "accompanying infection" is meant a purely tuberculous process in the lung, with a subsequent infection of some other portion of the lower air passages by another microbe or microbes. By an "intercurrent infection" is meant the occurrence of quite distinct acute disease in a patient suffering from pulmonary tuberculosis, this new disease running its own definite course, and producing its own clinical phenomena. I imagine that Dr. Habershon's view is a very similar one, his Class (*a*) cases being what Dr. Radcliffe would term a true secondary infection, and his Class (*b*) cases what we recognise as accompanying or intercurrent infections.

True secondary infections, in which the infection by the tubercle bacillus is complicated subsequently by the presence of one or more than one infecting organism, occur in our

[1] A paper read at a discussion at a Meeting of the Bristol Medico-Chirurgical Society on May 13th, 1914.

experience less commonly than is supposed. In an investigation of thirty-three cases associated with all the signs and symptoms generally recognised as evidence of a mixed infection, infection by tubercle alone was demonstrated in twenty-five. In the other cases the following secondary organisms were found, streptococcus mitior, the influenza bacillus, a Gram positive coccus, a staphylococcus, and a diplococcus. Vaccine treatment of cases of advanced pulmonary tuberculosis, complicated by a mixed infection, is not encouraging, and here again our experience seems to be similar to that of Dr. Habershon. In the first place it is very difficult to isolate the infecting organism, and in the second place the associated extensive tuberculosis in itself usually renders the outlook hopeless.

Dr. Radcliffe further made a search for evidence of any appreciable degree of septicæmia by an examination of the blood by cultural methods. Twenty-two cases of well-marked disease were thus examined, with one positive result. In this case the infecting organism was the staphylococcus albus, and the patient was successfully treated by a vaccine.

Record of the patient from whose blood a staphylococcus was obtained :—

Mr. C., age 23, admitted April, 1909. Duration of disease, three months. Type of case : Chronic pulmonary tuberculosis. (Group 2.) Right lung, infiltration of apex of lower lobe. Left lung, extensive disease of upper lobe, with some consolidation of apex of the lower lobe. General condition very fair. Appetite and digestion normal. Daily range of temperature 98° F. a.m. to 100° F. p.m. Pulse rate 80 to 90.

During the first five weeks of his stay in the sanatorium he was treated on the ordinary sanatorium lines, and was kept at rest practically throughout this period. During these five weeks he gained six pounds in weight, his temperature fell to a range of 98° F. to 99° F., and his pulse rate to 80–85. Examination of his chest at the end of his first month showed slight improvement in the physical signs, the lower lobe of the right lung being clear of moist sounds. On May 17th he had an acute exacerbation, his temperature running up to 103.4° F. On

May 19th a specimen of his blood was taken for bacteriological examination. Throughout the week of May 23rd to 29th his temperature remained almost constant at a range of 100° F. a.m. to 100.5° F. p.m., and he suffered much from the malaise commonly associated with this degree of fever.

*Bacteriological Examination.*—From each of five tubes inoculated with blood the same organism was obtained. This grew well on ordinary media and liquefied gelatine. Morphologically, staphylococcus albus. Although the possibility of contamination could not be absolutely excluded, it was decided to try the effect of vaccine treatment, especially as the staphylococcus obtained was not the ordinary staphylococcus epidermidis albus, and because the patient's opsonic index to the organism tended to be low. The reaction of the organism in Gordon's tests is given in table :—

| | Litmus Milk. | | Neutral-red broth. | Nitrite. | Maltose. | Lactose. | Glycerine. | Mannite. | Growth on gelatine. | Liquefaction of gelatine. | Gram's stain. |
|---|---|---|---|---|---|---|---|---|---|---|---|
| | Acid. | Clot. | | | | | | | | | |
| *a* | – | – | – | + | + | + | + | + | + | + | + |

Litmus milk became slightly more alkaline.

*Treatment.*—Was guided by frequent estimations of the opsonic index, and the initial doses were exceedingly small until the effect had been observed. The vaccine was given on May 28th, June 3rd, 8th, 16th, and 23rd. The immediate effect of the injection of the vaccine was a disappearance of the malaise. This was very marked, and the patient felt well and took his food better. With the subjective improvement was associated a fairly rapid fall in temperature. (*See chart, p.* 129.)

Examination on June 29th showed that there had been some extension of the disease, the right upper lobe having become affected. The lesions in the right lower lobe and in the left lung at that date were very similar to their condition on May 11th. No body weight was lost during these five weeks of fever. On July 15th another injection of vaccine was given. The patient's temperature at that time was fairly steady at a range of 97.6° F. a.m. to 99° F. p.m. Following the injection of the vaccine, the temperature immediately fell to a range of 97.6° F. to 98.2° F., and the patient made rapid improvement. On July 30th examination of his chest gave the following result :

Name M.C.

### Chart 7.

Shows the effect on the Opsonic Index of a number of injections of a Staphylococcus Vaccine made from an organism isolated from the blood stream.

### AVERAGE CHART FOR 7 DAY PERIODS.

Adventitious sounds still present in the upper and lower lobes of both lungs, but much less numerous and less moist in character; sputum reduced in amount by more than half during the past four weeks. General health good and improving, walking three miles daily. Since July 30th, patient has made steady and satisfactory progress. (*Average temperature chart.*)

The result has been of value, as the patient's temperature was gradually brought down, but before this occurred the effect on his general condition was marked. (*Chart* 7.)

After treatment had been continued for several months, a second blood culture was undertaken, and as this proved sterile, and as the patient by this time was taking hard exercise without any effect on his temperature, treatment was stopped.

The accompanying and intercurrent type of secondary infection, such as an infection of the air passages with M. catarrhalis, pneumococcus, etc., as Dr. Habershon has pointed out, offers more scope for successful treatment with vaccines. The offending organism is more readily recognised, and a hopeful factor is that the tuberculous process is often limited and quiescent in character. I believe that this type of secondary infection is less commonly met with amongst patients in sanatoria than amongst patients attending hospitals in large cities, the explanation being that continuous living in pure air tends to clear both pulmonary and laryngeal lesions of many secondary organisms. Prophylactic measures against these secondary infections are of importance, such as removal of septic teeth and eradication of any septic foci in the upper air passages.

### Tuberculin Treatment in Pulmonary Tuberculosis.

In the first place, we have to recognise that the theoretical grounds in support of tuberculin therapy are but slight. The experimental production of immunity to tuberculosis by inoculations of tuberculin has not been demonstrated; in fact, Austrian,[1] in a recent investigation, found that animals sensitised to tuberculin succumbed to infection by

[1] *Johns Hopkins Hosp. Bull.*, 1913, xxiv. 280.

tuberculosis as readily as control animals which had not received tuberculin. No protective or bactericidal properties have been demonstrated in the serum of patients or animals immunised to tuberculin. It is not surprising, then, that attempts to treat the disease on the lines of passive immunity by the use of sera have failed. The value of tuberculin therapy has to be judged by the results obtained in its clinical application. We must note, too, that since we have no certain knowledge of the action of tuberculin, its administration is a matter of empiricism, and in consequence a subject of keen controversy. At King Edward VII Sanatorium we now have nearly three years' continuous work with tuberculin behind us, and are in a position to form an estimate as to how far we have succeeded in obtaining the results claimed for it.

The advantages of treatment with tuberculin, as compared with treatment by general hygienic measures alone, are generally stated to be :—

(1) That in patients in which T.B. have not yet appeared in the sputum the percentage of cases which make a complete recovery amounts to nearly 100 per cent., and that subsequent relapses are prevented.

(2) That among patients suffering from early or moderately advanced disease, with T.B. in the sputum, the number of recoveries is greater when tuberculin is used.

(3) That the proportion of cases in which T.B. disappear from the sputum is appreciably increased when tuberculin is given, in addition to general measures of treatment.

Our experience on these points has been as follows :—

(1) That cases of pulmonary tuberculosis, in which T.B. are not found in the sputum, do equally well with or without tuberculin. For example, in 1907–8 120 cases of this type were treated in the Sanatorium, all of them being discharged apparently well. What is more to the point is, that at the present time, alter a lapse of from six to seven years, only seven deaths have occurred amongst these patients—one from insanity, one from cancer, and five from tuberculosis. The remainder are well. I have no reason to expect that the results of our cases of this kind treated with tuberculin will be any less satisfactory or any more so. There is indeed little scope for improvement.

It is clear that the treatment of these bacillus-free cases with tuberculin is uncalled for, and that in view of the good results obtained with simple hygienic measures the records of such cases treated with tuberculin can supply us with no useful information as to the value of this form of treatment.

With respect to the second point :—

(2) In 1912 and 1913 we treated 200 favourable cases of early and moderately advanced disease (all of which had T.B. in their sputum) with tuberculin. Our immediate results were : Arrested or much improved, 68 per cent. ; stationary or worse, 16 per cent. A satisfactory enough result. But our records for 1910–11, when no tuberculin was used, show that the results of treatment of 200 similar cases were precisely the same.

With regard to the third point :—

(3) As to disappearance of T.B. from the sputum. Of the 200 cases just referred to, which in 1912–13 were treated in the Sanatorium with tuberculin, 27 per cent. lost their bacilli before discharge ; but again, of the 200 cases of the same type treated without tuberculin in 1910–11, the percentage figure for loss of bacilli was almost identical, namely 27.5 per cent.

Further, with rigid selection of cases, excluding all save those with a limited apical lesion associated with complete absence of constitutional symptoms, the percentage figure for loss of bacilli worked out as high as 50 per cent., both in the case of patients treated with and without tuberculin. The selection of case was the favourable factor in each series.

Although excellent results may indeed be expected from treatment by tuberculin inoculations (providing that they are carefully administered, and reactions strictly avoided), combined with the general measures of treatment as carried out in a sanatorium in the case of patients suffering from pulmonary tuberculosis in its earlier stages, our experience does not suggest that these good results are the result of the inoculations, since when general measures alone are prescribed equally favourable results are secured, and with greater security from possible mishap.

The following passages from the well-known work by Bandelier and Roepke,[1] two of the ablest and most experienced advocates of tuberculin, is pertinent to our experience. The limitations of tuberculin could scarcely be better expressed :—

" Attempts to reduce fever are not suitable for ambulant practice. Harm will generally be done by tuberculin in the case of badly-nourished patients. If, on economical grounds, hypernutrition is impossible, or when attempted is unsuccessful, it is better to dispense with tuberculin. Where also there is no time for regular measurement of temperature, where a fresh-air cure by night and rest for a few hours daily is impossible, where hydro-therapeutic measures are impracticable, where, lastly, there is no sense of personal hygiene and prophylaxis, then tuberculin injections alone will not prevent the spread of the disease ; it is best, therefore, to spare oneself and others the disappointment which must follow the use of tuberculin."

This sound advice is unfortunately often unheeded; at the same time; when tuberculin is given under the conditions

[1] Bandelier and Roepke, *Tuberculin in Diagnosis and Treatment.* Seventh Edition. London : Bale Sons & Danielsson. 1913.

thus laid down by Bandelier and Roepke, conditions which have been proven to have a markedly beneficial effect on pulmonary tuberculosis, it is manifestly difficult to estimate how much, if any, of the benefit that follows is due to the inoculations.

In conclusion, I should like to quote my impressions of tuberculin as stated in an Interim Report recently submitted to the Consultant Medical Staff of the Sanatorium;

"Our experience is that tuberculin is not a remedial agent which can be depended upon to revolutionise either our sanatorium results or our conception of the outlook for the average consumptive. Tuberculin has not proved itself to be a remedy in the ordinary sense of the term, and no immediate or striking results are to be expected from it, even in the most favourable cases.

"Tuberculin, as employed in the Sanatorium, in association with the favourable conditions which are secured by residence in the institution, exercises an influence on those cases in which steadily-increasing doses can be taken. The treatment produces (1) tolerance to considerable doses of tuberculin, and in many cases (2) an effect on the diseased structures. This local effect may be a source of danger.

"As to the kind of case in which it may be expected that tuberculin treatment is at least without unfavourable effect, our experience is that it is the patient with a good outlook who has rapidly responded to general hygienic measures, and who has shown evidence of constitutional vigour and recuperative power.

"In a considerable number of cases tuberculin, so far as can be judged from immediate clinical results, has no obvious influence on the lesions. The patient is gradually made tolerant to it, but this seems to be the only objective indication to its effect. Whether this tolerance is of value is a point which has yet to be established.

"Further, in a proportion of cases tuberculin is not merely inert, it is definitely prejudicial.

"As far as the Midhurst results show, tuberculin cannot be looked upon as a means whereby an unfavourable case can be converted into a favourable one, or as likely to turn the scale in a patient's favour when his progress is doubtful, and certainly not when it is definitely retrogressive. More often than not in such cases it will do harm.

"Our experience shows that the administration of tuberculin is quite unsuitable as a routine method of treatment of pulmonary tuberculosis, and that its indiscriminate and careless use on a large scale can only end in harm."

These are not the conclusions which we had hoped to arrive at, and which personally I had anticipated, but our experience permits of none other.

Dr. LATIMER SHORT, County Tuberculosis Officer for Somerset, remarked that it had been very interesting to hear the varied experience of two such eminent observers working amidst different surroundings, and he noticed that Dr. Habershon appeared to have seen more striking results than Dr. Bardswell. This was quite parallel with his own experience in London and in Somerset, that dwellers in an urban district appeared to do better on vaccines than those in a rural district, presumably because the former were more liable to the influence of a mixed infection than the latter, and therefore the results of its specific treatment were more readily seen. Sanatorium routine itself treated the mixed infection, and a vaccine might not be so necessary as where less favourable conditions prevailed. He emphasised the necessity of first getting rid of any possible focus of infection, evidenced by oral sepsis, leucorrhœa or chronic tonsillitis, and quoted illustrative cases. Radical dental treatment was a very logical and important part of sanatorium benefit, and should be more frequently included in it. He mentioned that the Somerset Insurance Committee had recognised the fact in a very gratifying manner.

[1] *Preliminary Report on the Treatment of Pulmonary Tuberculosis with Tuberculin.* H. K. Lewis. 1914.

## Figures and Tables

**Chart 7. f1:**
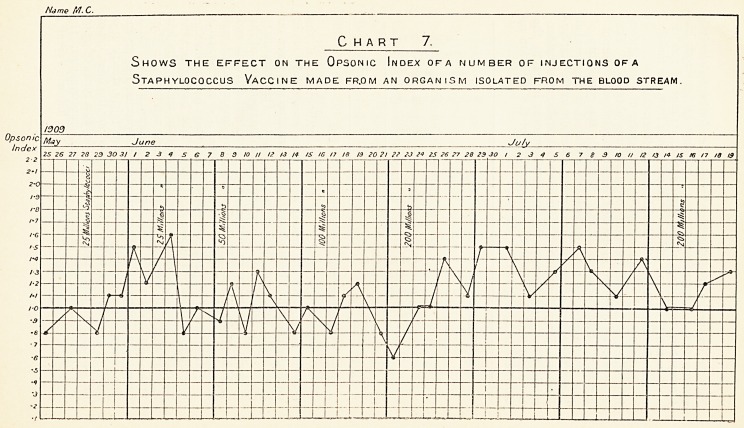


**Figure f2:**